# The Inquiline Ant *Myrmica karavajevi* Uses Both Chemical and Vibroacoustic Deception Mechanisms to Integrate into Its Host Colonies

**DOI:** 10.3390/biology10070654

**Published:** 2021-07-12

**Authors:** Luca Pietro Casacci, Francesca Barbero, Piotr Ślipiński, Magdalena Witek

**Affiliations:** 1 Department of Life Sciences and Systems Biology, University of Turin, Via Accademia Albertina 13, 10123 Turin, Italy; 2 Museum and Institute of Zoology, Polish Academy of Sciences, ul. Wilcza 64, 00-679 Warsaw, Poland; piotrslipinski@gmail.com (P.Ś.); mawitus@yahoo.co.uk (M.W.)

**Keywords:** host–parasite interactions, social parasitism, cuticular hydrocarbons, stridulation, *Myrmica* ants

## Abstract

**Simple Summary:**

Although fiercely defended by workers, ant societies can be infiltrated by social parasites that exploit the whole colony and its resources, instead of taking advantage of a single organism. Among them, we studied *Myrmica karavajevi*, a rare ant species in which the worker caste does not exist, and queens live as “inquilines” within colonies of another ant species, commonly *Myrmica scabrinodis*. Parasite queens entirely depend on host worker labour. They have evolved many adaptations to be admitted and integrate as members of the host ant society. Indeed, we found that, even at the pupal stage, the parasite is treated as a valuable item in the colony hierarchy, as it is rescued ahead of the colony’s own brood. Moreover, we discovered that *M. karavajevi* adult queens use two strategies to live undisturbed in the host nest. The parasite smells like the host queens by possessing specific odours and produces sounds similar to those emitted by *M. scabrinodis* ants, particularly by queens. Through these kinds of mimicking, *M. karavajevi* advances its rank towards the highest attainable position in the colony’s hierarchy. *M. karavajevi* is an outstanding example of multimodal deception, which needs a combined behavioural and molecular approach to be fully unravelled.

**Abstract:**

Social parasitism represents a particular type of agonistic interaction in which a parasite exploits an entire society instead of a single organism. One fascinating form of social parasitism in ants is the “inquilinism”, in which a typically worker-less parasitic queen coexists with the resident queen in the host colony and produces sexual offspring. To bypass the recognition system of host colonies, inquilines have evolved a repertoire of deceiving strategies. We tested the level of integration of the inquiline *Myrmica karavajevi* within the host colonies of *M. scabrinodis* and we investigated the mechanisms of chemical and vibroacoustic deception used by the parasite. *M. karavajevi* is integrated into the ant colony to such an extent that, in rescue experiments, the parasite pupae were saved prior to the host’s brood. *M. karavajevi* gynes perfectly imitated the cuticular hydrocarbon profiles of *M. scabrinodis* queens and the parasite vibroacoustic signals resembled those emitted by the host queens eliciting the same levels of attention in the host workers during playback experiments. Our results suggest that *M. karavajevi* has evolved ultimate deception strategies to reach the highest social status in the colony hierarchy, encouraging the use of a combined molecular and behavioural approach when studying host–parasite interactions.

## 1. Introduction

Species interactions, e.g., mutualism or parasitism, are pivotal driving forces in evolution. The key outcome of parasite-host coevolution processes are adaptations evolved by parasitic species and counter-adaptations arising as a response in hosts. The impact of parasites could be very high as they can influence the host’s life-history traits, fitness, behaviour, or sexual choices (e.g., [1]).

Colonies of social insects, e.g., bees, wasps, termites, or ants, are attractive targets for many species of parasite, like viruses, bacteria, nematodes, mites, as well as parasitic insects and numerous species of social parasites [2]. In a strict definition, the latter group encompasses a social species that exploits host colony resources as food, shelter or reproductive effort [3]. Social parasitism occurs in all groups of social insects, but it seems to be less common in termites and widespread in ants (e.g., [3,4,5]). There are various types of social parasitism in ants from temporary parasites to obligate inquilines, reflecting different levels of parasite’s host dependence [3]. Obligate inquilines are closely related to the host species (Emery’s rule [6]) with whom they share many life-history traits [7,8]. Following the terminology presented by Buschinger [3], inquiline ants are permanent parasites that are fully dependent on their hosts and, in most cases, lack a worker caste. Therefore, the inquiline queen relies on host workers and can invest all her resources to produce sexual forms [3]. Several inquiline species are host queen-tolerant, which means that they coexist inside the same colony together with host queen(s) and all of them can reproduce. Nevertheless, the majority of sexuals are usually produced by the inquiline queen [9].

As the fitness of inquilines is wholly dependent on the host work, they have to evolve various adaptations to enter and integrate within host colonies [10]. The most important capability is to break the ant chemical communication channel which is a sophisticated code used by colony members to recognise nest-mates from non-nestmates [11,12]. This code is created by cuticular hydrocarbons (CHCs) and differences in this chemical signature play the main role as recognition cues (e.g., [11,13]). Social parasites can use various chemical strategies to mislead their hosts: chemical mimicry (active production of CHCs), camouflage (acquisition of colony odour) or chemical insignificance (reduction of specific classes of CHCs not to be detected by hosts) [13,14,15].

Besides the chemical channel, social parasites can also exploit other hosts’ communication systems like visual and acoustical cues. The latter ones have recently been studied in myrmecophilous butterflies including the *Maculinea* genus that encompasses only obligate social parasites of *Myrmica* ants [16,17,18,19,20,21,22,23]. It was demonstrated that larvae and pupae of these butterflies are able to manipulate their *Myrmica* hosts by mimicking vibroacoustic signals to obtain a high status in the host colony hierarchy [17]. 

*Maculinea* butterflies are only one of the many organisms found in *Myrmica* nests. Inquilinism as well has evolved several times in this ant genus [24,25], which is, therefore, among the best-studied models to explore invertebrate symbiotic dynamics [26]. Notwithstanding, our knowledge about interactions between inquiline ants and their hosts and about parasite adaptations is still very limited, primarily because inquilines are rare and often found in isolated patches within their host range [3]. One of such extremely rare and poorly known ant species is *Myrmica karavajevi* (Arnoldi, 1930). More than 80 years after the species description, *M. karavajevi* was reported from less than 30 localities scattered throughout nearly 20 European countries [27,28,29]. The most common host of this inquiline ant is *M. scabrinodis* Nylander, 1846 [30], but *M. karavajevi* was also found inside colonies of four other *Myrmica* species, all belonging to the ‘*scabrinodis* group’ [27]. Usually, various host species are reported from different countries [24,27]. Results of a molecular phylogenetic study by Jansen et al. [25] revealed that *M. karavajevi* might be classified within the ‘*scabrinodis* group’ (i.e., together with its host species), suggesting the existence of a relatively recent common ancestor shared with its hosts, and representing Emery’s rule in the loose sense, which means it derives from the same genus to which its hosts belong [3]. However, this inquiline ant cannot be considered as a sister species of any other *Myrmica* species (for further discussion see [25]). 

Similar to many other inquilines of social insects, adaptations used by *M. karavajevi* to enter and integrate within host colonies are still unknown. The main aim of our study was to assess the level of parasite integration inside host colonies and to investigate if and how inquiline queens are able to deceive the multimodal communication channels of their hosts. Briefly, we found that inquiline immature stages are intimately integrated into the colony hierarchy to such an extent that host ants rescued *M. karavajevi* pupae in preference to their own brood. Besides, both chemical and acoustic communication channels of the host can be potentially corrupted by the inquiline queens (i) whose CHCs perfectly matched those of host queens, and (ii) which emit specific vibroacoustic signals partially resembling those of host queens and eliciting the same behavioural responses in the host workers as host queens.

## 2. Materials and Methods

### 2.1. Collection and Sample Maintenance

We sampled one population of *Myrmica scabrinodis* in Kraków, Poland (50°01′ N/19°53′ E). In the field, we searched for *Myrmica scabrinodis* nests. All found colonies were opened, and the brood examined in search for *Myrmica karavajevi* sexual pupae. At pupal stage, the parasite is easily detectable, being similar to the host workers in size but having wings. Parasitised colonies were excavated and brought to the laboratory. In the laboratory, we set up ant colonies of 100 workers in 28 cm × 15 cm × 10 cm^3^ Perspex containers and reared them on a diet of sugar and *Drosophila* larvae. 

### 2.2. Brood Rescue Experiment

For the brood-rescue experiment, we used Brian nests [31] consisting of two adjacent chambers whose internal dimensions are 8 × 4 × 0.75 cm^3^ and 8 × 2 × 0.75 cm^3^ communicating at one end. Three *Myrmica scabrinodis* sclerotised worker pupae, three white gynes pupae and three *Myrmica karavajevi* white gyne pupae were randomly located on a 0.4 cm^3^ moist sponge (to preserve humidity) at the end of one compartment, which was then covered with transparent glass. Nine *M. scabrinodis* workers were then placed in the chamber together with the colony brood. We covered the other chamber with a dark glass. After the workers had rested for 10 min in the dark, we shone a 60 W light placed 10 cm away onto the chamber containing the worker ants and brood, to create a high level of stress which induced workers to rescue the exposed brood items and carry them into the dark chamber. We recorded the order and the time at which each item of brood was rescued. We performed three replicates for each of the three *M. scabrinodis* colonies used for testing the rescue behaviour and using different workers and brood. Therefore, a total of 81 brood items (27 for each of the three categories) was tested.

### 2.3. Chemical Analysis

The analyses of CHCs were performed using the protocol described by Csata et al. [32]. Briefly, 7 *M. scabrinodis* queens, 5 males, 7 workers and 7 *M. karavajevi* queens and 5 males belonging to three colonies were placed in separate clean glass vials and their CHCs were solvent-extracted using 200 μL of hexane for 20 min. The extracts were then stored at −20 °C until analysis. Prior to chemical analysis, 800 ng of n-eicosane (*n*-C20) were used as an internal standard. After evaporation under a nitrogen flow to achieve a final volume of 20 μL of heptane, we analysed 2 μL of each sample in an Agilent 7890B gas chromatograph coupled with an Agilent 7000C mass spectrometer using a Gerstel MPS autosampler. For the technical description of the GC, the program of temperatures used as well as the mass spectra setting refer to Csata et al. [32]. We analysed mass spectra by compiling previous publications and assessing fragmentation by comparing patterns obtained from the injection of standard series of n-alkanes. We manually integrated the chromatograms to calculate the area of each peak using the proportion of the sum over the area of all peaks. The absolute quantity of CHCs (ng) per ant was measured as the sum of all the peaks area divided by the peak area of the internal standard (*n*-C20) and multiplied by 800 (ng of the internal standard used per each sample). The resulting amount of each CHC was divided by the individual’s surface area in square millimetres to account for the different sizes of host and parasite castes. For the calculation of the surface area, we took pictures of individual ants using a Leica EZ4 W stereomicroscope. Then, the ant bodies were ideally divided into geometric areas and their dimensions were measured using the software ImageJ 1.53a (see [33] for a detailed description of the method).

### 2.4. Recording of Vibroacoustic Signals

After one week of settlement in laboratory conditions, we recorded the vibroacoustic emissions of individual workers (*n* = 7) and queens (*n* = 5) of *Myrmica scabrinodis* and *M. karavajevi* queens (*n* = 5) from three colonies. Recordings were collected with a custom recording equipment described in detail by Riva et al. [34] consisting of a 12.5 × 8 × 2 cm^3^ recording chamber with a moving-coil miniature microphone (sensitivity: 2.5 mV/Pa/1.0 kHz) attached through the centre (sampling rate set to 44.10 kHz). A second identical moving-coil microphone was used to record in anti-phase the ambient noise. A mixer and output amplifier (dynamic range: 5 Hz to 40 kHz; gain: 53 dB) combined the signals from the two microphone preamplifiers. Overall, the frequency ranged from 20 Hz to 20 kHz and the gain was approximately 83 dB. The equipment was powered by a 12 V gel cell battery, and the recording chamber and microphones were located inside an anechoic chamber to further reduce ambient noise and interference.

Ants were individually placed on the microphone surface within the recording chamber and recorded in the morning at room temperature (23–25 °C). Each ant was recorded for 10 min, starting 5 min after its introduction into the recording chamber.

Segments containing vibroacoustic recordings were carefully inspected, checked for clipping and digitally saved in WAV format (16-bit amplitude resolution) on a computer using Audacity v. 2.2.2 [35]. The temporal and spectral features of the signals were measured using Praat v. 6.1.05 [36].

We then randomly selected 4–7 trains per individual consisting of a variable number of pulses and measured for each pulse 15 temporal and spectral parameters (Supplementary Table S1). We then computed a pairwise Spearman rank correlation analysis on the 15 vibroacoustic parameters using R v. 4.0.3. From a pair of parameters with r_s_ > 0.7, only one was selected for analysis. This method yielded seven vibroacoustic variables: the third quartile of the energy spectrum (Q75%, Hz; 75% of the call energy); the frequency peak (Fpeak, Hz); the frequency standard deviation (SDQ50, Hz); the relation of the frequency peak energy to the call total energy expressed as a percentage (%EFpeak); the mean intensity of the entire call represented by the root-mean-square signal level (RMS, dB), the pulse duration (Δt, s), and the pulse rate (PR, s^−1^; calculated as 1/t_start_(x) − t_start_(x + 1)).

### 2.5. Scanning Electron Microscopy

Six host queens and workers and five parasite queens were dissected between the postpetiole and the abdomen to reveal the stridulatory organ (SO) made of the pars stridens and the plectrum. The head and the two ant parts of each individual were mounted on the same steel stub and coated with gold, and the samples were scanned using a Cambridge Stereoscan S360 scanning electron microscope. The SEM operated at 20–25 kV.

We then measured the length (maximum distance between the basal and the apical ridge bisecting the organ in two equal parts) and width (maximum ridge length) of the pars stridens using the software ImageJ 1.53a [37] and counted the number of ridges present on the pars stridens per 100 μm.

### 2.6. Playback

Playback assays were carried out in three 7 × 7 × 5 cm^3^ artificial arenas with the speaker attached at the bottom of the box as described in detail by Sala et al. [19]. We covered the speaker with a thin layer of slightly humid soil. Ten host workers from the same *M. scabrinodis* colony were positioned in each arena and allowed to settle for 10 min before being played one of four vibroacoustic signals (*M. scabrinodis* queen, *M. scabrinodis* worker, *M. karavajevi* queen, and white noise as control). We used MP3 devices to play loops of the original recordings, adjusting volumes to the natural level (see Sala et al. [21] for detail). Each trial lasted 40 min: counts were made of all instances of antagonistic and attractive behaviours, during periods of one minute for each box, and in sequence between the four treatments, i.e., 10 min in total for each signal per trial. Each playback experiment was repeated 9 times, using fresh ants from three different *M. scabrinodis* colonies (i.e., three times for each colony), therefore a total of 360 workers was tested. The source of signals for each arena was randomised to control for possible positional effects. New soil was introduced, and all the equipment was cleaned with absolute alcohol between trials.

### 2.7. Statistical Analysis

Statistical analyses were performed with the software R v.4.0.0 [38].

Brood rescue data were analysed using the R package “coin”. Kruskal–Wallis tests were used to compare the rescue order and time (s) of different brood categories. Subsequent pairwise comparisons of median rescue order and time (s) between brood categories were made using Wilcoxon–Mann–Whitney tests. *p* values were calculated against a null distribution generated from data using a Monte Carlo resampling.

Partial least squares discriminant analysis (PLS-DA), implemented in the “mixOmics” R package [39], was used to determine differences or similarities between members of different castes based on their CHC profiles [40,41]. The PLS-DA is a supervised pattern recognition technique which is largely unaffected by co-variance among chemicals and small ratio between cases (samples) and variables (chemicals) [42,43]. In addition, the predict function was used to carry out a full cross validation (leave-one-out) test based on Mahalanobis distance, thus obtaining a contingency table (confusion matrix) of posterior-predicted/observed membership of all analysed samples. We reiterated this operation 999 times and calculated the average (±SE) percentages of assignment for each caste. Discriminant cuticular hydrocarbons responsible for chemical profile variation were identified according to their influence on the projection (VIP) parameter [44]. We also calculated Euclidean distances between *M. karavajevi* and *M. scabrinodis* queens on the basis of log-transformed data of epicuticular hydrocarbon concentrations (ng/mm^2^). The intra- and inter-colonial distances were then compared using the Welch Two Sample *t*-test after checking for normality.

We draw a non-metric multidimensional scaling ordination (NMDS) based on Euclidean distances using the R package “vegan” [45] on the normalised vibroacoustic parameters of signals emitted by the recorded specimens. To test for differences in vibroacoustic signals among the host (queens and workers) and parasite (queens) castes, we conducted a permutational multivariate analysis of variance with 999 permutations using the nonparametric adonis2 function implemented in the R package “vegan”. Vibroacoustic parameters were compared among castes using a linear model approach (LM, maximum likelihood fit). Tukey’s HSD test was used to calculate post-hoc comparisons on each factor in the model using the function glht from the R package “multcomp” [46].

Finally, the effect of the vibroacoustic stimulus on the sum of all worker ant behaviours and on each separate behaviour was analysed in a linear mixed-effects model with “colony” as a random factor. Post-hoc sequential comparisons among factor levels were carried out using the lsmeans function from the “lsmeans” R package [47]. A Wilcoxon-Mann-Whitney Test was used to assess differences in the SOs of host queens and workers and parasite queens.

## 3. Results

### 3.1. Social Status of Parasite and Host Brood

**Figure 1:**
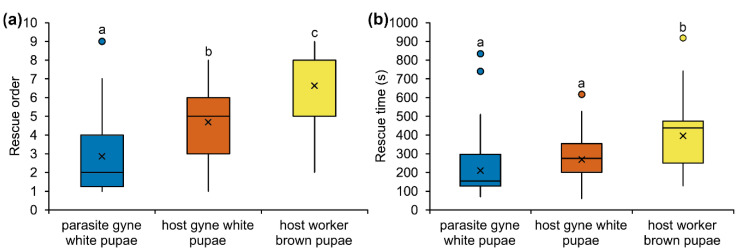
Boxplots illustrate (**a**) the order and (**b**) the time (s) in which *M. scabrinodis* worker ants rescued *M. karavajevi* queen unsclerotised (white) pupae, host queen unsclerotised (white) pupae and host worker sclerotised (brown) pupae, after their nests were perturbed by exposure to light. Horizontal line = median rank of rescue, x = average value; box = 25th–75th percentiles; whiskers = minimum and maximum values; dots = outliers. Different letters indicate significantly different categories based on Wilcoxon-Mann-Whitney Test (Supplementary Table S2).

In the rescue assays (Figure 1a; Supplementary Table S2), *M. karavajevi* white pupae were saved ahead of *M. scabrinodis* queen white pupae (Mann-Whitney test: U = −2.363, *p* = 0.018) and worker sclerotised pupae (U = −4.004, *p* < 0.001). Among the colony brood, the worker sclerotised pupae were the last to be rescued, significantly behind the host queen unsclerotised (white) pupae (U = −2.716, *p* = 0.007). When we considered the rescue time (Figure 1b; Supplementary Table S2), *M. scabrinodis* workers took the same time to rescue the parasite and the host queen white pupae (U = −1.3058, *p* = 0.198) but the removal of the workers sclerotised pupae took longer compared to the other brood items (compared with parasite queen white pupae—U = −2.995, *p* = 0.003; compared to host queen white pupae—U = −2.1701, *p* = 0.031).

### 3.2. Chemical Integration

Gas-chromatography analysis revealed a total of 45 cuticular hydrocarbons present on the cuticle of the analysed specimens (Appendix A, Table A1). The branched alkanes represented the most abundant hydrocarbon class (21 peaks of which 15 monomethylated and six dimethylated alkanes), followed by alkenes (13 peaks, of which nine monoenes and four dienes) and linear alkanes (11 peaks). Overall, five peaks (*n*-C23, *n*-C25, 3-MeC23, as well as one diene and one monoene both constituted by 25 carbons) were present in a high proportion, ranging from about 65% in the case of *M. scabrinodis* queens, up to about 90% in the workers of the same species (Appendix A, Table A1). Interestingly, specimens of host and parasite queens showed the most complex cuticular profiles constituted by the same number (45) of hydrocarbons (Appendix A, Table A1).

**Figure 2:**
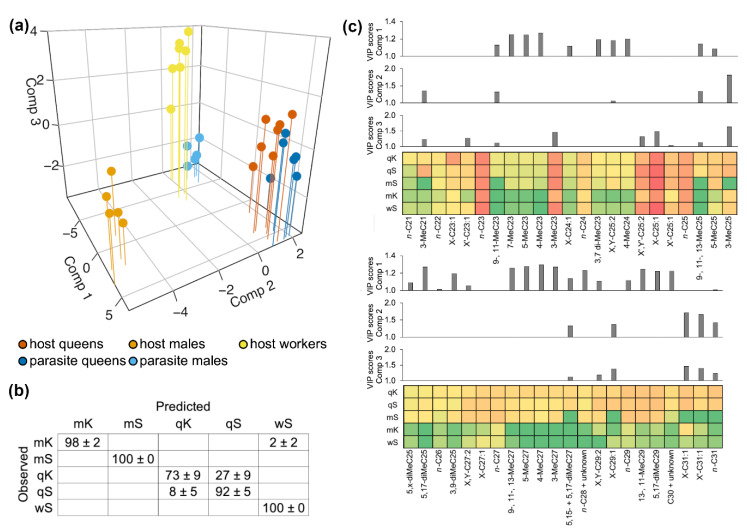
(**a**) Representation of standardized components 1, 2 and 3 of partial least squares discriminant analysis for log-transformed concentrations (ng/mm^2^) of cuticular hydrocarbons found on the cuticle of parasite and host castes (qS = *M. scabrinodis* queen; mS = *M. scabrinodis* males; wS = *M. scabrinodis* workers; qK = *M. karavajevi* queen; mK = *M. karavajevi* males). The first three components accounted respectively for the 61%, 13% and 9% of the total variation. (**b**) Below, the contingency table showing percentages (± SE) of assignment for each caste after full cross validation tests is reported (qS = *M. scabrinodis* queen; mS = *M. scabrinodis* males; wS = *M. scabrinodis* workers; qK = *M. karavajevi* queen; mK = *M. karavajevi* males). (**c**) For each of the three components obtained from the PLS-DA the variable importance in projection (VIP) score plot is reported. Below the VIP score plots, the colored squares (green = minimum values; red = maximum values) represent the average log-transformed concentrations (ng/mm^2^) of the corresponding compounds present on the cuticle of each caste.

PLS-DA on the log-transformed concentrations (ng/mm^2^) of cuticular hydrocarbons well separated *M. scabrinodis* worker and male specimens and *M. karavajevi* males (Figure 2a). On the contrary, host and parasite queen individuals partially overlapped. Full cross validation confirmed these observations with *M. scabrinodis* workers and males and *M. karavajevi* males showing 98–100% of specimens being correctly assigned while host and parasite queens having lower percentages (see contingency table in Figure 2b). *M. karavajevi* queens were mistaken with higher percentages for host colony queens (27% ± 9%) while *M. scabrinodis* queens were confused with the parasite queens in 8% ± 5% of cases. If we consider the queens (of the host and the parasite) as a whole, the error rate can reach 35% ± 14%, i.e., which in the most extreme cases translates into a misattribution percentage of 50% between host and parasite.

Overall, the first three components accounted for 83% of CHC variation. The first component contributed to separate the specimens in two groups, one including the host and parasite queens and the host males while the second including the host workers and parasitic males (Figure 2a; see also Supplementary Figure S1 where only the first two axes are represented). The variable importance plots (VIP, Figure 2c) showed that numerous branched alkanes contribute the most to explain the separation in groups observed on the first axis. On the second axis, it is worth noting the clear separation of *M. scabrinodis* males which have different concentrations in some methylated alkanes, e.g., 3-MeC21 and 3-MeC25 and long-chain monoenes, e.g., C29: 1 and C31: 1.

It is also interesting to note how the concentrations of some linear alkanes, e.g., *n*-C21, *n*-C22, *n*-C23, *n*-C24, *n*-C25, are very similar between hosts and parasites, having VIP scores below 1. Some of these linear compounds, e.g., *n*-C23 and *n*-C25, constitute a large portion of the CHC profile, with maximum values in some castes equal to 30% in the case of *n*-C23 and 18% for *n*-C25.

Finally, when we compared Euclidean distances based on chemical profiles of parasite queens and host colony queens with the distances between parasite and non-host colony queens, we found no significant differences (*t*-test: t = −0.91522, df = 23.025, *p* = 0.3696).

### 3.3. Vibroacoustic Integration

#### 3.3.1. Vibroacoustic Signals

**Figure 3:**
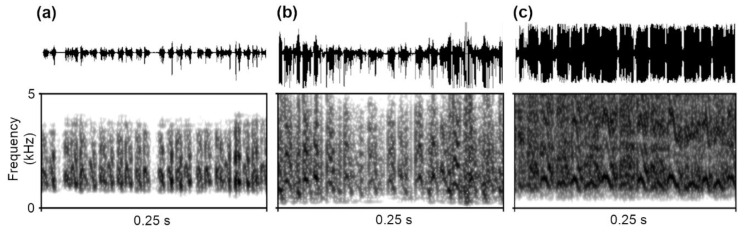
Oscillograms and spectrograms of the stridulations emitted by (**a**) *M. karavajevi* queens and (**b**) *M. scabrinodis* queens and (**c**) *M. scabrinodis* workers. Spectrograms were generated in Praat using the following parameters: window shape = Gaussian, window length = 0.015 s, number of time steps = 1000, number of frequency steps = 500, dynamic range = 70 dB.

We recorded and analysed the stridulation produced by *M. scabrinodis* workers and queens and from *M. karavajevi* queens, for a total of 1204 pulses. Ants’ stridulation patterns were similar to those described in previous works carried out on the genus *Myrmica* [17,21,48]. The stridulations were constituted by series (trains) of a variable number of pulses (Figure 3).

**Figure 4:**
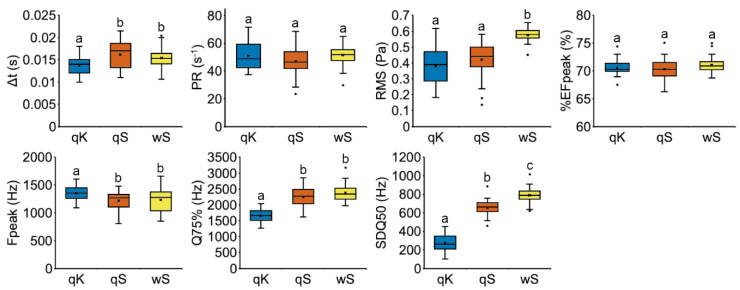
Boxplots of vibroacoustic parameters of stridulation pulses emitted by *M. scabrinodis* queens (qS), workers (wS) and *M. karavajevi* queens (qK). Pulse duration (∆t), pulse repetition frequency (PR), root-mean-square (RMS), percentage of energy (%EFpeak), frequency peak (Fpeak), third frequency quartile (Q75%), frequency standard deviation (SDQ50). Horizontal lines represent median values, the boxes the first and third quartiles, whiskers maximum and minimum values, while the crosses indicate average values. Dots represent outliers. Lower-case letters above boxplots indicate pairwise significant differences between castes based on Tukey’s HSD test (Supplementary Table S3).

Our data showed that the vibroacoustic signals produced by the host queens and workers have similar temporal and frequency characteristics (Figure 4; Supplementary Table S3), i.e., the duration of the pulses (∆t), the frequency peak (Fpeak) and the third frequency quartile (Q75%), which differ from the signals emitted by *M. karavajevi* queens. The peak frequency is higher in the pulses produced by the parasitic queens, but the higher range of frequencies seems to be generally lower than in host signals (consider the third frequency quartiles). Nevertheless, some characteristics, i.e., the pulse repetition frequency (PR) and the percentage of energy (%EFpeak) allocated for the emission of the frequency peak, were similar between host and parasite ants (Figure 4). In addition, the root-mean-square (RMS) signal level resulted identical between the signals of the host and parasite queens, while the frequency standard deviation (SDQ50) differs among the three categories (qK, qS, wS; Figure 4).

Non-metric multidimensional scaling ordination carried out on the vibroacoustic parameters of pulses shows overall separation among the three categories (host queen and worker, and parasite queens), confirmed by the permutational multivariate analysis of variance (F_2,112_ = 26.344; *p* = 0.0001). The signals emitted by *M. karavajevi* queens were statistically different from those emitted by both *M. scabrinodis* workers (F_1,78_ = 51.765; *p* = 0.0001) and (F_1,78_ = 23.261; *p* = 0.0001) queens. Nevertheless, the parasite signals were closer to those of the host queens than to those emitted by the worker caste of the colony. Moreover, the overall vibroacoustic signal is different between host queen and workers (F_1,78_ = 9.497; *p* = 0.0001).

#### 3.3.2. Playback Assays

During playback experiments, worker ants did not exhibit any antagonistic or alarmed behaviour (alerting) but four benevolent responses were observed, i.e., walking, antennating, staying on the speaker, and digging.

The observed behaviours involve attraction or benevolent reactions: (1) walking—the worker was attracted to the speaker but walked over it without stopping on it; (2) staying—the workers rested on the speaker for at least 5 s; (3) antennating—the worker antennated the speaker for at least 3 s; (4) digging—the worker dug into the soil surrounding the speaker. The response called repulsion (the ant abruptly changed direction, moving away from the speaker) was observed only when the control stimulus (white noise) was played back to workers.

**Figure 5:**
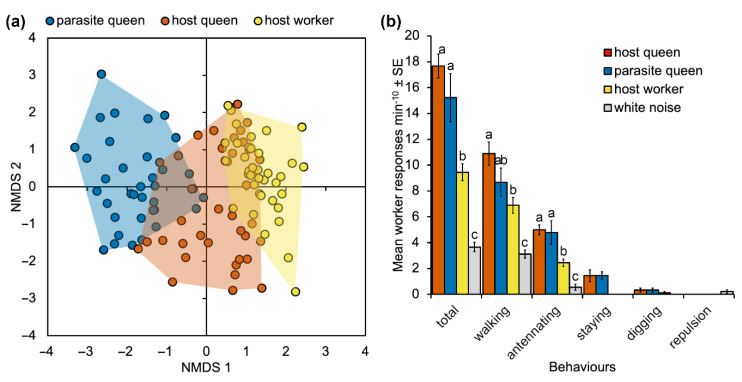
(**a**) Non-metric multidimensional scaling ordination of vibracoustic signals emitted by *M. scabrinodis* workers and queens and *M. karavajevi* queen ants based on normalized Euclidean distances calculated using pulse parameters. Each point represents ‘average’ values of pulse parameters calculated over a train of pulses (see “Methods” for more details) (STRESS = 0.18). (**b**) Behavioral responses of *Myrmica scabrinodis* workers to vibroacoustic emissions of *M. scabrinodis* queens and workers, *M. karavajevi* queens and to one control signal (white noise). Lower-case letters above columns indicate pairwise significant differences between vibroacoustic stimuli based on Tukey’s HSD test (Supplementary Table S4).

Generalized linear models showed that worker reactions to the four vibroacoustic stimuli were significantly different for walking, antennating, staying and for the total behaviours. Digging behaviour, which was observed on a few occasions, was never elicited by white noise (Figure 5b; Supplementary Table S4). Compared to white noise, ant vibroacoustic signals always induced significantly more instances of walking, antennating, and staying by *Myrmica* worker ants (0.019 < *p* < 0.0001). Both vibroacoustic emissions by host and parasite queens elicited the highest behavioural response in worker ants for walking, antennating, and staying, while no significant difference between the two stimuli was detected for any behaviour (Figure 5b; Supplementary Table S4).

#### 3.3.3. Stridulatory Organs (SO)

**Figure 6:**
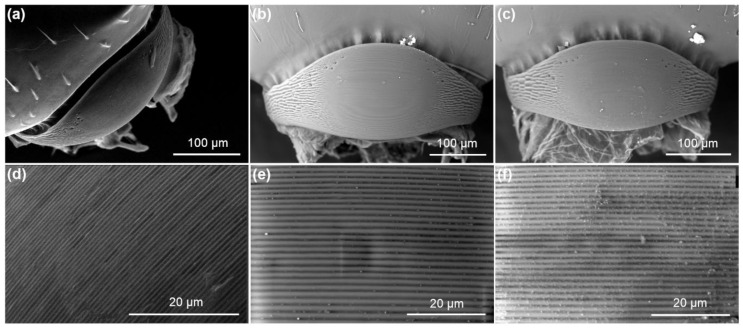
Electron Scan Microscope images of the whole *pars stridens* and ridges of (**a**,**d**) *Myrmica karavajevi* queens and (**b**,**e**) *M. scabrinodis* queens and (**c**,**f**) workers.

The SO of *M. karavajevi* is described for the first time within the present work (Figure 6). Overall, it is very similar to the organ present in all other species of the genus *Myrmica* studied so far [16,17,21,48,49].

**Figure 7:**
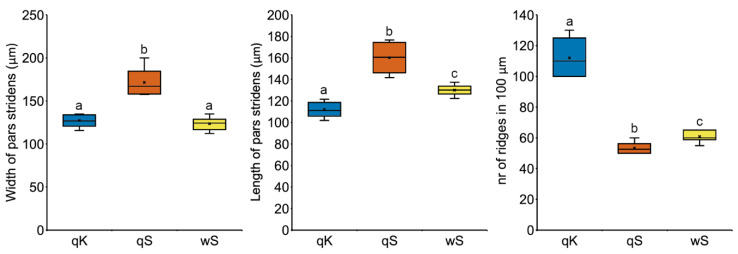
Boxplots of the main characteristics measured on the stridulatory organs of the three categories, i.e., the width and length of the *pars stridens* and the number of ridges distributed along 100 μm (see Section 2.5 for details). Horizontal line = median rank of rescue, box = 25th–75th percentiles; whiskers = minimum and maximum values; crosses = average values. qK = parasite queen; qS = host queen; wS = host worker. Different letters indicate significantly different categories based on Wilcoxon-Mann-Whitney Test (Supplementary Table S5).

However, *M. karavajevi* SO differs in some morphological characteristics from the SOs of the host castes. In detail, the pars stridens in *M. karavajevi* is smaller (width and length) than that of *M. scabrinodis* queens (Figure 7). In contrast, the pars stridens width is similar in *M. karavajevi* and host ant workers, but the latter possess a longer organ (Figure 7; Supplementary Table S5).

This similarity in the pars stridens to the host worker caste is explained by a similar body size of *M. karavajevi* queens to the adult workers. Despite this, the ridge density of the parasite’s pars stridens is much greater than that of the two host castes’ organs (Figure 7; Supplementary Table S5).

Furthermore, *M. karavajevi* SO differs in form. If we compare the ratio between its width and height among the three categories (parasite queen, host queens and workers), this value is statistically different (Kruskal–Wallis test: H = 6.393, df = 2, *p*-value = 0.041). The organ of the parasite has an elongated shape, i.e., the median value of the ratio between width and length is equal to 1.147 and is statistically different (U = 2.556, *p*-value = 0.0092) from the ratio measured on the host workers (median value = 0.934). The SO of host queens, on the other hand, has an almost circular shape with a median value of the ratio between width and length equal to 1.079, which does not differ from *M. karavajevi* (U = 0.912, *p*-value = 0.426), nor from workers (U = 1.4412, *p*-value = 0.179).

## 4. Discussion

Our results show that *Myrmica karavajevi* is intimately integrated within the colonies of its host *M. scabrinodis*. To achieve its acceptance within host nests, the parasite has intercepted in the course of evolution the chemical cues to fully integrate into the colonies and the acoustic signals to gain the worker’s attention and protection. The inquiline species seems to occupy the highest place in the hierarchy of the colony, a condition found in the immature stages, which are saved by host ants in preference to their own brood in rescue experiments.

Ant brood, by using different cues, contains information about development stage, caste, sex or species and adult workers can recognise it [50]. Social parasites, at which brood parasitism occurs, are under the pressure to avoid host detection and evolve strategies allowing brood to survive and develop and be treated by host workers as their own larvae and pupae. It is known that ants, as the first action after nest disturbance, rescue their brood and that the rescue order depends on social hierarchy among the different young stages, reflecting colony investment in each of these stages [48,51]. Therefore, the results of our rescue experiment suggest that *M. karavajevi* pupae achieve the highest rank in the colony hierarchy and they are fully integrated in the colony structure as they were the first ones taken by host workers, even before host queen pupae. As inquilinism in ants is rare and at least studied in the sense of host–parasite coevolution [3], there is no other data on ant inquilinism system to compare with. By contrast, the ecology of inquilinism was more extensively studied in parasite wasps (Hymenoptera: Vespidae) [11] and results of some studies on this insect group show that socially parasitic brood can reach high integration and rank status inside host colony. It was demonstrated that larvae of *Polistes sulcifer* (Zimmermann, 1930), the obligate social parasite of *Polistes dominula* (Christ, 1791) paper wasp, can monopolise host parental care and grow much faster compared to the host brood [52]. The acceptance and integration of parasite immature brood was also found in the case of *Maculinea* butterflies, which are obligate social parasites of *Myrmica* ants. In the rescue experiments *M. rebeli* (Hirschke, 1904) larvae, just after one week of integration inside the host colony, were chosen equally with the kin pupae of *Myrmica* host and significantly ahead of kin larvae [53].

Such high parasitic brood integration inside a host colony can be achieved by using various adaptations. Our results demonstrate the ability of *M. karavajevi* to break multiple communication channels of their host. The comparison of CHCs among castes revealed that *M. karavajevi* queens possess a complex CHC profile that is qualitatively identical to that of the host queens. Although differences in the abundances of each compound exist among castes, contributing to separating them, the chemical profile of the parasitic queens also partially overlaps with that of the host queens.

Imitation of the epicuticular profile of the host colony represents a common strategy among social parasites to overcome the efficient nestmate recognition system which acts as a barrier in eusocial insect colonies, and successfully penetrate the host fortress. Nestmate recognition is usually based on a complex mixture of hydrocarbons covering the insect’s cuticle, which primarily prevent water loss and the entry of pathogens [54], but it also plays an important role in communication, creating a bouquet of substances that characterise each colony [55].

Social parasites can imitate the host recognition code through chemical mimicry, in which the parasite actively synthesises host CHC signals, and the mimetic profile may also be the result of both passive and active acquisition of CHC compounds from the host, a strategy named camouflage [56,57,58]. Instances of camouflage were found in *Temnothorax minutissimus* (Smith, 1942) ants, which acquire the odour of their *Temnothorax curvispinosus* (Mayr, 1866) host queens by active grooming [59]. Camouflage is also found in queens of a slave-making ant species such as *Polyergus samurai* Yano, 1911, when usurping *Formica japonica* Motschoulsky, 1866 host nests. *p. samurai* queens obtain a colony-specific signature, which resulted in being much more similar to the chemical profile of the host queen killed. The latter similarity is likely achieved by body contact [60]. This kind of chemical strategy is largely employed in non-ant myrmecophilous species. Among other examples, larvae of a lycaenid butterfly, *Niphanda fusca* (Bremer & Grey, 1853) exploits *Camponotus japonicus* Mayr, 1866 ants by acquiring male chemical profiles [61]. However, Hojo and colleagues [61] did not exclude that some methyl-branched compounds typical of male ants can be actively synthesised. Thus, the two chemical strategies (chemical mimicry and camouflage) could occur contemporarily in the same biological system.

Evidence of chemical mimicry is scarce and primarily found in species-specific associations. The biosynthesis of mimetic compounds has been suggested in *Leptothorax kutteri* Buschinger, 1966 to enhance the exploitation of *L. acervorum* (Fabricius, 1793) host ants [62]. The majority of studies highlighting the existence of chemical mimicry involved myrmecophilous species other than ants, such as lycaenids of the genus *Maculinea*. These larvae gain access to the host colony resources by the active production of mimetic compounds. *M. rebeli* caterpillars synthesise CHCs which are peculiar of their primary host ants *Myrmica schencki* Viereck, 1903, but which would pinpoint the parasite as a “stranger” in other *Myrmica* species. Thus, their biosynthesis is basically suppressed when *M. rebeli* is adopted in non-host *Myrmica* nests.

Parasites also use a third strategy, chemical insignificance, which is based on a general reduction or a scanty presence of informative CHCs (i.e., *n*-alkanes) as respectively shown in *Brachymyrmex* thief ants [11] and in *Acromyrmex insinuator* Schultz et al., 1998 exploiting its host *A. echinatior* (Forel, 1899) [63,64].

To understand whether mimetic compounds are biosynthesised or acquired, parasites are usually experimentally isolated from the host colony for a certain time, so that they can reveal their own CHC profile [65,66,67]. In our experiment, we could not do so due to the paucity of sampled nests and the analysed individuals. However, we cannot exclude both strategies to be adopted by the parasite. Indeed, we can hypothesise that *M. karavajevi* queens may acquire their mimetic CHC profiles living inside the host colonies, coming into contact with the host individuals, and being fed by the workers of the host colony. On the other hand, one clue that may suggest the active biosynthesis of mimetic compounds is related to the size of the parasite. Indeed, the body surface area (see Supplementary Table S6) of parasitic queens is equal to half the body surface (40.8 mm^2^ ± 3.5 mm^2^) of *M. scabrinodis* queens (84.7 mm^2^ ± 5.1 mm^2^) and similar to that of the host workers (43.0 mm^2^ ± 6.1 mm^2^). The fact that the amount (ng) of hydrocarbons per area is similar between parasitic and host queens suggests that the parasite may be able to synthesize double (or nearly double) amounts of hydrocarbons. It is unlikely for the parasitic queens to acquire such amounts of hydrocarbons by contact, and it is equally unlikely that from simple trophallaxis, used in ant colonies to exchange food or colony odours, queens can then extrude or heavily spread epicuticular compounds on their cuticle in such an amount to mimic the host queens. In addition, when we tested if the CHC profiles of inquilines follow those of the hosts at the colony-level (see Section 3.2), by comparing the Euclidean distances based on the CHC profiles of *M. karavajevi* queens with host queens from the same colony and different colonies, we did not find any difference. These results suggest that all inquiline queens are chemically similar to host queens in general, but with no obvious differences among colonies, hence pointing towards the mimicry strategy for which the CHC similarity is innate.

However, there are still many doubts about how the recognition process works in eusocial insects. For instance, it is not clear whether some signals are more informative than others and whether the simple presence/absence of one or more compounds can be decisive in the recognition of nestmates or whether CHC concentrations are also used for discriminating between nestmate and non-nestmates [68,69]. Some works have emphasized the quality of cuticular hydrocarbons by attributing different importance for the purposes of intra- and interspecific communication to the three classes of hydrocarbons, i.e., linear alkanes, branched alkanes, and alkenes. In certain experiments, the supplementation of linear alkanes to non-nestmates does not cause a defensive reaction in the individuals of the host colony, while the alkanes and branched alkenes do [70,71]. Furthermore, the greater the quantity of these complex hydrocarbons, the stronger the defensive reaction of the hosts becomes [72]. Recently, however, the role of linear alkanes has been revised in another key. In colonies of *M. scabrinodis*, infected with the ectoparasitic fungus *Rickia wasmannii* Cavara, 1899, infected individuals showed a percentage increase of some linear alkanes, i.e., *n*-C23 and *n*-C24, and the same individuals were attacked less than uninfected individuals in which the epicuticular profile was unchanged [32]. At the same time a decrease of another methylated alkane was also observed, i.e., 3-MeC23, which has suggested that the linear CHC and its methylated counterpart may be part of a recognition mechanism that, once discovered by some social parasites, may have favoured the integration and exploitation of *Myrmica* colonies.

In our study, the queens of the parasite have high percentage levels of *n*-C23 (15.81 ± 5.31) and lower percentage levels of the methylated counterpart, i.e., 3-MeC23 (8.37 ± 1.98). It is therefore possible that the parasite, besides having developed an overall similarity towards the host queen CHC profile, may rely on only some compounds for its deception system, perhaps linear alkanes, whose increase seems to have the ability to reduce the aggressiveness in the workers of *M. scabrinodis*. This fact appears to be even more evident for the parasitic males that besides possessing CHC profiles, closer to that of the workers of the colony (Figure 1a), reach percentage levels of *n*-C23 equal to 30% which, in addition to those of *n*-C25, constitute half of their epicuticular profile. Such a strategy could be beneficial for the parasite males, who find themselves flickering inside the host colony, and must avoid being attacked by the *M. scabrinodis* nurses with the brood chambers.

Besides an efficient imitation of the overall chemical profile of their hosts (or the use of certain compounds such as linear alkanes) to fool or appease *Myrmica* ants, *M. karavajevi* queens are also able to break a second communication code by mimicking the host queen’s stridulations.

Indeed, vibroacoustic communication is extensively used by ants from different subfamilies, e.g., Myrmicinae, Ponerinae [73], both inside and outside their nests (see [74] for a review), therefore it is likely that intruders might produce signals directed to disrupt this type of communication. This ability has been observed in a few other social parasites such as butterflies [17,21,74] and beetles [75]. An increasing body of evidence suggests that vibroacoustic emissions are not only attractive cues but rather are used to signal the social rank within the colony, at least in some Myrmicinae ants. The studies performed on species belonging to *Myrmica* [17] and *Pheidole* [75] genera reveal that ants are capable of producing caste-specific vibroacoustic signals, which can elicit several distinct responses in nest-mates.

The analysis of similarities performed on the vibroacoustic signal emitted by different *M. scabrinodis* castes and *M. karavajevi* queens (Figure 5) confirms that host queens and workers overall produce distinct sounds and reveals that the parasite signals better resemble those of host queens rather than workers. The ability to produce signals matching those of the highest rank caste can be partially explained by the morphology of the stridulatory organ (SO). The pars stridens shape of *M. karavajevi* SO is much more similar to host queen SO than to workers SO, although the ridge numbers strongly differ among the three categories (host queens and workers and parasite queens). The width and height of the SO differs between host and parasitic queens due to the size of the body, but the shape, described by the relationship between width and height, is similar. On the contrary, the shape of the SO of the queens of *M. karavajevi* and the host workers is different, but the individual of these two castes have the same body size. If we compare the morphology with the characteristics of the signals produced, the difference in the number of ridges, higher in the parasitic queens, could explain the diversity of the frequency parameters found in the analysis of the acoustic emissions.

However, it is still unknown whether ants perceive the whole vibroacoustic signal or if one or few components may be more informative than others [34]. In our behavioural experiments, we played back the full repertoire of the whole recordings, thus assessing the overall effect of acoustic emissions on the ant colony. During playback experiments, we found that the queen-like sounds produced by the parasite and the queen host vibroacoustic emissions cause the highest rate of benevolent response in workers, exactly as observed for *Maculinea* butterfly parasite [17]. Unlike what is known in the latter host-parasite systems, the information available on the life cycle of *M. karavajevi* is scanty. Therefore, we do not know if adult stages of the parasite are indeed treated like royalty in the host ant colony, as it happens for *Maculinea* larvae in *Myrmica* nests [65].

*M. karavajevi’*s ability to break both the chemical and acoustic code of the host is not surprising. Indeed, there is a close phylogenetic relationship between the parasite and the host species. In some cases, host and parasite are closely related sister species and follow the strict Emery’s rule, while in other cases they belong to the same genus, following the relaxed version of Emery’s rule, as happens in the case of *M. karavajevi* and *M. scabrinodis*.

In addition, *M. karavajevi* can use several host species and some studies have hypothesized that some sort of local sympatric speciation has occurred within populations [25,76,77], which led to the formation of cryptic genetic lines, each specialized on a particular species of *Myrmica* of the *scabrinodis*-group, which all colonize warm but relatively humid environments [27].

The close phylogenetic origin of many obligate social parasites with their hosts indicates that they share many traits of evolutionary history and have developed strategies to efficiently exploit the host system, deriving from ancestral phenotypic traits [8,10,78]. Thus, such examples are particularly valuable for exploring the mechanistic basis, such as molecular regulatory processes, of social traits that are subtly (but measurably) different between host and parasite. When closely related species of parasites and hosts are compared, it is difficult to assess if the similarity in certain phenotypic traits, e.g., CHCs or vibroacoustic parameters, is due to adaptation or to common ancestry. Some hints could be provided by comparing the chemical profile of another member of the *scabrinodis*-group, *M sabuleti* Meinert, 1861 [79]. As *M. karavajevi* shares a common ancestry with both *M. scabrinodis* and *M. sabuleti*, we could hypothesise that a higher chemical similarity of the parasite to its host might be due to the coevolutionary dynamics rather than to phylogenetic closeness. In the work by Guillem and colleagues [79], indeed, the CHC compounds used to differentiate *M. scabrinodis* and *M. sabuleti*, these two closely related species, are two methylated alkanes, i.e., 3-MeC23 and 5-MeC25, present in greater proportions only in one of the two species, respectively. If we compare the relative proportions reported in Guillem et al. [79] with our data, *M. karavajevi* queens possess the 3-MeC23, the characteristic compound of *M. scabrinodis*, in higher proportions (10%), while the relative proportion of 5-MeC25, characteristic of *M. sabuleti,* is close to 1%, suggesting a closer adaptation towards *M. scabrinodis*. However, this remains to be formally tested. As suggested by Jansen et al. [25], *M. karavajevi* may even represent a complex of cryptic species, each adapted to a specific host ant, but a comparison between multiple host and parasite populations would be necessary.

Overall, the results from rescue experiments suggest that *M. karavajevi*, at least as gyne pupae, achieves a high social status in the colony hierarchy. However, further research is needed to unravel if this royal treatment is also gained by the adult parasite queens and obtained through a supernormal stimulus (sensu Tinbergen [80]), as our acoustical and chemical data seem to suggest.

## 5. Conclusions

A plethora of distinct signals (chemical, vibroacoustic, tactile and visual) concur to a fine multimodal communication in ants, which is at the base of the complex social structure of the colony. Nevertheless, we have poor knowledge about how parasites might manipulate ant responses and behaviours by means of multiple cues. It is likely that chemical and vibroacoustic communications have evolved independently, but in strictly obligate parasites these two channels may have started to function in a multimodal way to let the parasite achieve the highest level of integration within the colony.

## Data Availability

The data supporting the findings of this study are openly available in the GitHub repository at: https://github.com/lcasacci/Casacci_et_al_2021_kara.git (accessed on 22 June 2021).

## Supplementary Materials

The following are available online at https://www.mdpi.com/article/10.3390/biology10070654/s1, Figure S1: Representation of standardized components 1 and 2 of partial least squares discriminant analysis for log-transformed concentrations (ng/cm^2^) of cuticular hydrocarbons, Table S1: List of the 15 vibroacoustic parameters, Table S2: Results of Kruskall-Wallis test and Wilcoxon-Mann-Whitney Test on rescue order and time (s) of brood items within *Myrmica scabrinodis* colonies, Table S3: Results of linear models on the vibroacoustic parameters, Table S4: Results of generalized linear models of *M. scabrinodis* worker responses to different vibroacoustic stimuli, Table S5: Results of Kruskall-Wallis test and Wilcoxon-Mann-Whitney test on the main features of the stridulatory organs, Table S6: Mean (± SD) values of body surface (mm^2^) for each caste.

## Appendix A

**Table A1:** Relative proportions ± SD of each cuticular hydrocarbon constituting the profile of *Myrmica scabrinodis* queens, workers and males and *M. karavajevi* queens and males. Compounds reported in bold contribute overall to a large proportion of the cuticular profile.

		*Myrmica scabrinodis*			*Myrmica karavajevi*	
nr	CHC	Queens	Workers	Males	Queens	Males
1	*n*-C21	0.14 ± 0.11	0.12 ± 0.08	0.06 ± 0.01	0.42 ± 0.15	0.12 ± 0.04
2	3-MeC21	5.64 ± 5.22	0.11 ± 0.13	---	0.71 ± 0.16	0.14 ± 0.09
3	*n*-C22	0.28 ± 0.1	0.3 ± 0.08	0.35 ± 0.11	0.64 ± 0.18	0.32 ± 0.09
4	X-C23:1	0.72 ± 0.19	0.55 ± 0.33	0.86 ± 0.3	7.28 ± 1.72	1.1 ± 0.15
5	X’-C23:1	0.85 ± 0.26	0.67 ± 0.34	0.87 ± 0.14	1.02 ± 0.14	0.16 ± 0.1
6	*n*-C23	8.4 ± 2.63	19.43 ± 7.17	11.76 ± 1.11	15.81 ± 5.31	30.93 ± 4.91
7	9-, 11-MeC23	0.2 ± 0.08	---	---	0.3 ± 0.06	---
8	7-MeC23	0.19 ± 0.08	0.02 ± 0.01	0.22 ± 0.1	0.28 ± 0.07	0.02 ± 0.01
9	5-MeC23	0.17 ± 0.11	0.02 ± 0.01	0.25 ± 0.15	0.31 ± 0.06	0.02 ± 0.01
10	4-MeC23	0.17 ± 0.08	---	0.16 ± 0.13	0.27 ± 0.05	---
11	3-MeC23	9.93 ± 1.63	14.2 ± 6.14	12.78 ± 1.92	8.37 ± 1.98	8.06 ± 1.04
12	X-C24:1	0.18 ± 0.09	0.03 ± 0.03	0.16 ± 0.12	0.27 ± 0.11	0.04 ± 0.02
13	*n*-C24	0.83 ± 0.16	1.07 ± 0.53	1 ± 0.16	1.52 ± 0.41	1.83 ± 0.53
14	3,7 di-MeC23	0.29 ± 0.11	0.07 ± 0.04	0.23 ± 0.12	0.47 ± 0.09	0.04 ± 0.03
15	X,Y-C25:2	0.41 ± 0.15	0.05 ± 0.02	0.66 ± 0.17	0.39 ± 0.08	0.02 ± 0.01
16	4-MeC24	0.3 ± 0.06	0.06 ± 0.04	0.31 ± 0.17	0.44 ± 0.08	0.04 ± 0.02
17	X’,Y’-C25:2	11.66 ± 9.84	9.99 ± 4.83	6.13 ± 1.6	3.84 ± 3.05	4.09 ± 1.62
18	X-C25:1	30.97 ± 8.84	42.77 ± 10.37	37.45 ± 5.28	21.66 ± 4.89	26.16 ± 3.7
19	X’-C25:1	2.12 ± 0.54	2 ± 1.15	2.64 ± 0.62	1.82 ± 0.69	1.32 ± 0.4
20	*n*-C25	4.8 ± 1.54	5.19 ± 3.99	7.18 ± 1.06	5.69 ± 1.06	17.59 ± 4.68
21	9-, 11-, 13-MeC25	0.56 ± 0.26	---	---	1.19 ± 0.53	---
22	5-MeC25	1.17 ± 0.27	0.32 ± 0.19	1.34 ± 0.31	1.26 ± 0.41	0.25 ± 0.15
23	3-MeC25	2.1 ± 0.56	1.28 ± 0.75		1.9 ± 0.68	2.31 ± 0.67
24	5,x-diMeC25	0.29 ± 0.1	0.07 ± 0.06	0.53 ± 0.36	0.36 ± 0.1	0.04 ± 0.02
25	5,17-diMeC25	0.56 ± 0.27	---	0.52 ± 0.17	0.41 ± 0.16	---
26	*n*-C26	0.37 ± 0.13	0.1 ± 0.08	0.34 ± 0.23	0.63 ± 0.16	0.13 ± 0.09
27	3,9-diMeC25	0.32 ± 0.18	0.01 ± 0.01	0.86 ± 0.58	0.57 ± 0.22	0.02 ± 0.01
28	X,Y-C27:2	1.13 ± 0.59	0.16 ± 0.1	1.16 ± 0.3	0.94 ± 0.62	0.15 ± 0.19
29	X-C27:1	1.1 ± 0.39	0.54 ± 0.51	1.13 ± 0.16	1.18 ± 0.54	0.26 ± 0.23
30	*n-*C27	0.87 ± 0.22	0.31 ± 0.25	1.54 ± 0.51	1.24 ± 0.46	0.8 ± 0.42
31	9-, 11-, 13-MeC27	0.57 ± 0.21	0.02 ± 0.01	0.83 ± 0.42	1.06 ± 0.56	---
32	5-MeC27	0.43 ± 0.11	---	0.78 ± 0.36	1.15 ± 0.57	---
33	4-MeC27	0.42 ± 0.08	---	0.53 ± 0.28	0.93 ± 0.26	---
34	3-MeC27	0.5 ± 0.18	---	0.96 ± 0.55	0.93 ± 0.31	---
35	5,15- + 5,17-diMeC27	0.74 ± 0.22	---		0.97 ± 0.24	---
36	*n*-C28 + unknown	0.32 ± 0.12	---	0.77 ± 0.38	0.73 ± 0.22	0.02 ± 0.01
37	X,Y-C29:2	1.27 ± 0.7	---	0.99 ± 0.54	1.1 ± 0.42	0.31 ± 0.12
38	X-C29:1	1.17 ± 0.36	0.12 ± 0.09	---	1.01 ± 0.32	0.02 ± 0.01
39	*n*-C29	1.09 ± 0.24	0.15 ± 0.11	1.59 ± 0.56	1.89 ± 0.73	0.53 ± 0.21
40	13-, 11-MeC29	1.35 ± 0.23	0.05 ± 0.04	1.57 ± 0.51	1.72 ± 0.61	0.08 ± 0.06
41	5,17-diMeC29	1.44 ± 0.34	0.04 ± 0.04	1.22 ± 0.36	1.77 ± 0.53	0.25 ± 0.19
42	*n*-C30 + unknown	0.54 ± 0.15	0.02 ± 0.02	0.27 ± 0.23	0.92 ± 0.34	0.03 ± 0.01
43	X-C31:1	1.7 ± 1.15	0.11 ± 0.12	---	1.76 ± 0.67	2.54 ± 1.89
44	X’-C31:1	0.66 ± 0.15	0.04 ± 0.04	---	1.1 ± 0.47	0.25 ± 0.08
45	*n*-C31	1.08 ± 0.43	0.03 ± 0.04	---	1.77 ± 0.51	0.02 ± 0.01
