# Supplementary material for: The Inquiline Ant Myrmica karavajevi Uses Both Chemical and Vibroacoustic Deception Mechanisms to Integrate into Its Host Colonies
Source: Biology (Basel). 2021 Jul 12;10(7):654. doi: 10.3390/biology10070654 (PMC8301377; doi:10.3390/biology10070654)
Supplement: Supplementary file 1 [file biology-10-00654-s001.zip › biology-1248881-supplementary.pdf]

# The inquiline ant *Myrmica karavajevi* uses both chemical and vibroacoustic deception mechanisms to integrate into its host colonies

**Luca Pietro Casacci [1,2,*], Francesca Barbero [1,*], Piotr Ślipiński [2] and Magdalena Witek [2]**

## Supplementary material

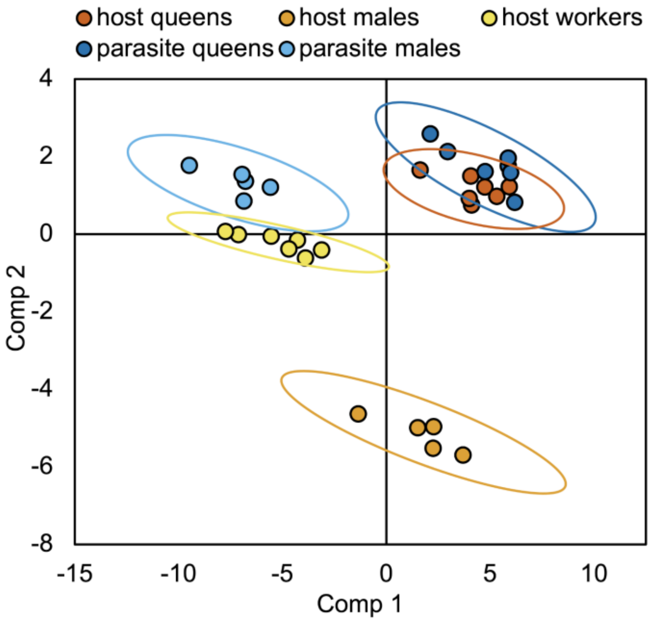


**Supplementary Figure 1.** Representation of standardized components 1 and 2 of partial least squares discriminant analysis for log-transformed concentrations (ng/cm$^2$) of cuticular hydrocarbons found on the cuticle of parasite and host castes (qS = *M. scabrinodis* queen; mS = *M. scabrinodis* males; wS = *M. scabrinodis* workers; qK = *M. karavajevi* queen; mK = *M. karavajevi* males). The two components accounted respectively for 61%, 13% of the total variation.

**Supplementary Table 1.** List of the 15 vibroacoustic parameters measured on the pulses, i.e., call fundamental unit, produced by each individual ant.

| Vibroacoustic parameters | Description | Unit |
|---|---|---|
| Q25% | Lower quartile of the energy spectrum | Hz |
| Q50% | Central quartile of the energy spectrum | Hz |
| Q75% | Higher quartile of the energy spectrum | Hz |
| SDQ50 | Frequency standard deviation | Hz |
| Fpeak | Frequency peak | Hz |
| EFpeak | Energy of the frequency peak | $Pa^2 s^{-1}$ |
| %EFpeak | Relation of the frequency peak energy to the call total energy | % |
| A | Mean amplitude | dB |
| P | Power | $dB^2$ |
| E | Energy | $Pa^2 s^{-1}$ |
| I | Intensity value | dB |
| RMS | Root-mean-square signal level | Pa |
| Δt | Duration of the pulse | s |
| PR | Pulse Rate, calculated as 1/tstart(x)-tstart(x+1)) | s-1 |
| CG | Centre of gravity | Hz |

**Supplementary Table 2** Results of Kruskall-Wallis test and Wilcoxon-Mann-Whitney Test on rescue order and time (s) of brood items within *M. scabrinodis* colonies. PQWP = parasite queen white pupae; HQWP = host queen white pupae; HWBP = host worker brown pupae.

| **Variables** | **Kruskall-Wallis test** | **Rescue order** | **Mann-Whitney Test** | **qKWP - qSWP** | **qKWP - wSBP** | **qSWP - wSBP** |
|---|---|---|---|---|---|---|
| **Rescue order** | H | 18.9850 | U | -2.3635 | -4.0042 | -2.7165 |
| | p-value | <0.0001 | p-value | **0.0178** | **<0.0001** | **0.0073** |
| **Rescue time** | H | 10.1350 | U | **-1.3058** | **-2.9948** | **-2.1701** |
| | p-value | 0.0063 | p-value | **0.1982** | **0.0031** | **0.0307** |

**Supplementary Table 3.** Results of linear models on the vibroacoustic parameters calculated on the stridulations emitted by *M. scabrinodis* queens and workers and *M. karavajevi* queens. Likelihood ratio (LR) statistics and results of Tukey multiple comparisons are reported for the delta time (Δt, s), pulse rate (PR, $s^{-1}$), root-mean-square signal level (RMS, Pa), third frequency quartile (Q75%, Hz), the frequency standard deviation (SDQ50, Hz), frequency peak (Fpeak, Hz), the relation of the frequency peak energy to the call total energy expressed as a percentage (%EFpeak, %). qS = *M. scabrinodis* queen; wS = *M. scabrinodis* worker; qK = *M. karavajevi* queen queen.

| | | LR | Tukey | qS - qK | wS - qS | wS - qK |
|---|---|---|---|---|---|---|
| **Δt** | Chisq | 17.021 | t value | 4.131 | 1.247 | 2.901 |
| | p-value | **0.0002** | p-value | **<0.001** | 0.4283 | **0.0125** |
| **PR** | Chisq | 5.4498 | z value | -1.782 | 2.187 | 0.375 |
| | p-value | 0.0655 | p-value | 0.181 | 0.078 | 0.925 |
| **RMS** | Chisq | 68.727 | z value | 1.811 | 7.316 | 9.028 |
| | p-value | **<0.0001** | p-value | 0.171 | **< 0.0001** | **< 0.0001** |
| **Q75%** | Chisq | 105.06 | z value | 10.161 | 2.068 | 12.201 |
| | p-value | **<0.0001** | p-value | **< 0.0001** | **0.101** | **<0.0001** |
| **SDQ50** | Chisq | 229.53 | z value | 19.490 | 6.831 | 26.228 |
| | p-value | **<0.0001** | p-value | **< 0.0001** | **< 0.0001** | **< 0.0001** |
| **Fpeak** | Chisq | 13.275 | z value | -3.434 | 0.488 | -2.953 |
| | p-value | **0.0013** | p-value | **0.0024** | 0.8773 | **< 0.0001** |
| **%EFpeak** | Chisq | 4.8531 | z value | -0.803 | 2.175 | 1.342 |
| | p-value | 0.0883 | p-value | 0.702 | 0.080 | 0.375 |

**Supplementary Table 4.** Results of generalized linear models of *M. scabrinodis* worker responses to different vibroacoustic stimuli. Likelihood ratio (LR) statistics and results of Tuckey multiple comparisons are reported for the sum of positive behaviours (total) and each behaviour separately (walking, antennating, staying). qS = host queen; qK = parasite queen; wS = host worker; WN = white noise.

| | | LR | Tuckey | qS - qK | qS-wS | qS-WN | qK-wS | qK-WN | wS-WN |
|---|---|---|---|---|---|---|---|---|---|
| **total** | Chisq | 104.4400 | z value | 1.278 | 4.661 | 8.22 | 3.457 | 7.341 | 4.613 |
| | p-value | **<0.0001** | p-value | 0.56768 | **< 0.001** | **< 0.001** | **0.00284** | **< 0.001** | **< 0.001** |
| **walking** | Chisq | 43.7570 | z value | 1.504 | 2.821 | 5.846 | 1.349 | 4.65 | 3.491 |
| | p-value | **<0.0001** | p-value | 0.42853 | **0.02389** | **< 0.001** | 0.52522 | **< 0.001** | **0.00255** |
| **antennating** | Chisq | 45.6760 | z value | 0.213 | 2.751 | 4.661 | 2.557 | 4.554 | 2.991 |
| | p-value | **<0.0001** | p-value | 0.9963 | **0.0273** | **<0.001** | **0.0469** | **<0.001** | **0.0132** |
| **staying** | Chisq | 36.0440 | z value | 0 | 0.004 | 0.004 | 0.004 | 0.004 | 0 |
| | p-value | **<0.0001** | p-value | 1 | 1 | 1 | 1 | 1 | 1 |

**Supplementary Table 5.** Results of Kruskall-Wallis test and Wilcoxon-Mann-Whitney test on the main features of the stridulatory organs of host and parasite castes, i.e. the width and height of the pars stridens and the number of ridges counted along 100 μm. qS = host queen; qK = parasite queen; wS = host worker.

| **Variables** | **Kruskall-Wallis test** | **Rescue order** | **Mann-Whitney Test** | **qK - wS** | **qS - wS** | **qK - qS** |
|---|---|---|---|---|---|---|
| **Width of pars stridens (μm)** | H | 11.359 | U | 0.91287 | 2.8823 | -2.7386 |
| | p-value | 0.003414 | p-value | 0.4256 | **0.0022** | **0.0028** |
| **Length of pars stridens (μm)** | H | 14.235 | U | -2.7386 | 2.8823 | -2.7386 |
| | p-value | 0.0008107 | p-value | **0.0055** | **0.0017** | **0.0035** |
| **nr of ridges along 100 μm** | H | 13.069 | **U** | 2.7767 | -2.4031 | 2.7767 |
| | p-value | 0.001453 | p-value | **0.0014** | **0.0274** | **0.0041** |

Supplementary Table 6. Mean (± SD) values of body surface ($mm^2$) for each caste (for details about calculation of body surface see von Beeren *et al.* 2011).

| **Body surface ($mm^2$)** | ***M. karavajevi* queens (N=7)** | ***M. karavajevi* males (N=5)** | ***M. scabrinodis* queens (N=7)** | ***M. scabrinodis* males (N=5)** | ***M. scabrinodis* workers (N=7)** |
|---|---|---|---|---|---|
| **Mean (± SD)** | 40.8 ± 3.5 | 35.2 ± 2.7 | 84.7 ± 5.1 | 77.9 ± 0.5 | 43.0 ± 6.1 |

## References


von Beeren, C.; Schulz, S.; Hashim, R.; Witte, V. Acquisition of chemical recognition cues facilitates integration into ant societies. BMC Ecol. 2011, 11, 1–13. doi.org/10.1186/1472-6785-11-30. doi.org/10.1186/1472-6785-11-30.
